# Metrnl/Meteorin-like/IL-41 Alleviates Rheumatoid Arthritis Via PPARγ-Mediated Suppression of Inflammation, Angiogenesis, and Bone Destruction

**DOI:** 10.1007/s10753-025-02426-x

**Published:** 2026-01-06

**Authors:** Tao Sun, Liping Xia, Yuxuan Li, Min Zhao, Zhuoqi Li, Hui Shen

**Affiliations:** 1 Department of Rheumatology and Immunology, The First Hospital of China Medical University, China Medical University, Shen Yang, 110002 China; 2 Department of Rheumatology and Immunology, Shengjing Hospital of China Medical University, China Medical University, Shen Yang, 110004 China; 3 National Clinical Research Center for Laboratory Medicine, Department of Laboratory Medicine, The First Hospital of China Medical University, Shenyang, 110002 China; 4 Department of Rheumatology and Immunology, China Medical University, Shen Yang, China

**Keywords:** Metrnl, Rheumatoid arthritis, Inflammation, Cytokine, Angiogenesis

## Abstract

Rheumatoid arthritis (RA), an autoimmune disease, is characterized by synovial hyperplasia, vascular occlusion, and bone erosion. Metrnl, a novel secreted protein linked to inflammatory immune regulation, has been implicated in RA pathogenesis, but its precise mechanisms remain undefined. This study aimed to elucidate Metrnl’s role in RA progression and therapeutic potential. Proteomic analysis was employed to assess Metrnl’s direct effects on RA fibroblast-like synoviocytes (RA-FLS). In vitro, LPS-induced RA-FLS were treated with Metrnl to evaluate proliferation, apoptosis, cell cycle progression, and expression of inflammatory cytokines (IL-6, IL-17, TNF-α) and angiogenic factors (PDGF, VEGF) via PPARγ signaling. Collagen-induced arthritis (CIA) mice models were established to validate therapeutic efficacy, with Micro-CT and histology quantifying joint damage and inflammation. Proteomics results indicated Metrnl’s multidirectional role in coordinating vascular homeostasis and immune-inflammatory network activation. Molecular biological results showed that Metrnl suppressed proliferation, promoted apoptosis, and downregulated IL-6, IL-17, TNF-α, PDGF, and VEGF through PPARγ in LPS-induced RA-FLS cells. In CIA mice, Metrnl mitigated weight loss, reduced swollen joints, and improved behavioral scores. Micro-CT confirmed attenuated cartilage/bone destruction and joint deformities, while histology revealed diminished inflammatory infiltration. Metrnl exerts anti-inflammatory and anti-angiogenic effects in RA by modulating PPARγ signaling, highlighting its dual role in suppressing synovitis and vascular remodeling. These findings propose Metrnl as a novel therapeutic target to impede RA progression, offering insights into its pathological mechanisms. Furthermore, Metrnl mitigates bone erosion and joint deformities, underscoring its broader translational potential for treating bone-related disorders.

**Supplementary Information:**

The online version contains supplementary material available at 10.1007/s10753-025-02426-x.

## Introduction

Rheumatoid arthritis (RA) is an autoimmune inflammatory disease characterized by synovial hyperplasia, vascular pannus formation, and cartilage damage, ultimately leading to joint deformity [1]. The imbalance of synovial fibroblasts and immune cells leads to the stimulation of pro-inflammatory cytokines and local tissue destruction [2, 3]. Controlling inflammation suppression and regression is of great significance for achieving clinical relief. The autoimmune attack on the immune system leads to the thickening of synovial fibroblasts and subsequent invasive tissue damage to bone and cartilage, while angiogenesis plays a driving role in early inflammation and synovial invasion. Therefore, it is crucial to explore small molecule therapeutic targets that can simultaneously inhibit both inflammation and angiogenesis in RA.

Metrnl is a newly secretory protein involved in inflammation and immune regulation [4, 5]. It is primarily secreted by adipocytes and activated macrophages, exhibiting high expression levels in mucosal barrier tissues, adipose tissue, skin, and muscle [6, 7]. Currently, research on Metrnl in inflammatory and immune diseases remains limited, mainly focusing on diabetes, asthma, inflammatory bowel disease and myocardial infarction [8, 9]. Metrnl’s initial exploration in the field of immunity stems from a research team’s discovery that Metrnl was highly expressed in RA synovium based on their own database of RA synovium. In Metrnl^−/−^ gene knockout mice, inflammatory lesions have been observed in both the skin and various internal organs, indicating that Metrnl may be involved in modulating inflammatory responses [10].

Metrnl is implicated in various human autoimmune diseases. Given its abundant presence in barrier tissues such as skin keratinocytes and fibroblasts, many studies have reported that Metrnl was highly expressed in dermatological conditions, including psoriasis and atopic dermatitis [11, 12]. Besides, serum Metrnl levels were elevated in patients with ulcerative colitis (UC) and Crohn’s disease (CD), correlating with inflammatory cytokines and disease activity indices [13]. Additionally, Metrnl has been detected in the intestinal mucosa of CD patients; systemic administration of Metrnl into an IL-10-deficient colitis animal model can improve disease progression while maintaining intestinal antibacterial peptide levels [14]. These findings suggested that Metrnl was involved in autoimmune responses. Recently, our previous study found that serum Metrnl level was increased in RA compared with healthy control and positively correlated with disease activity [15]. Therefore, Metrnl may play a regulatory role in the pathogenesis of RA; however, the specific mechanism remains unclear.

Considering that current biologics and small-molecule inhibitors for RA achieve sustained remission in only 30–40% of patients and carry a high risk of infection as well as a substantial financial burden, there is an urgent need for alternative therapeutic targets [16, 17]. Metrnl is an endogenous, pleiotropic secreted factor with both immunomodulatory and metabolic functions [18], making it a promising candidate to transcend the limitations of single-pathway inhibitors. Although one study has shown that Metrnl suppresses inflammatory responses by activating PPARγ [19], its role in RA has not yet been investigated, and it remains unclear whether any potential benefits in RA would indeed be PPARγ-dependent. In this study, to preliminarily explore the effects of Metrnl on human RA synovial cells and to support future research, total Metrnl-treated RA fibroblast-like synoviocytes (RA-FLS) protein was extracted for quantitative protein sequencing. Based on the analysis results, we further explored the mechanisms by which Metrnl modulates inflammation and vascular homeostasis. To simulate an inflammatory environment in synovium cells, we employed lipopolysaccharide (LPS)-stimulated human RA-FLS cells. Subsequently, we investigated the effects of Metrnl stimulation on LPS-induced RA-FLS cells, assessing parameters including proliferation, apoptosis, cell cycle progression, and the secretion of inflammatory cytokines and angiogenesis-related factors. Recent studies have demonstrated that Metrnl ameliorates the inflammatory response in LPS-stimulated human umbilical vein endothelial cells via PPARγ pathway, suggesting that Metrnl’s immunoregulatory role in RA may also involve PPARγ-dependent mechanisms. To test this hypothesis, we further examined whether Metrnl exerts its protective effects through regulation of PPARγ. Furthermore, we established a collagen-induced arthritis (CIA) mouse model to evaluate the therapeutic efficacy of Metrnl treatment in vivo.

## Methods

### Cell Preparation and Culture

Human RA-FLS cells obtained commercially (Shanghai Honsun Biological Technology, China) were maintained in fibroblast-specific medium (Sciencell, USA) containing 2% FBS, 1% fibroblast growth supplement, and 1% penicillin/streptomycin. RA-FLS cells between passages 2–4 were utilized to ensure phenotypic stability, with medium renewal every 48 h and culture conditions maintained at 37 °C with 5% CO₂.

### Quantitative Proteomics and Bioinformatics Analysis

Total cells were collected from RA-FLS after Metrnl-treated 48 h and sent for quantitative proteomics analysis to Hangzhou Jingjie Biotechnology Co., Ltd. (Hangzhou, China). The experiment group was treated with 50 ng/mL recombinant METRNL protein, while the control group received a blank treatment. Each group included three biological replicates.

Processed proteomics data underwent systematic analysis through the following workflow: Raw data preprocessing included protein database searching, peptide quality assessment (segment filtering and length evaluation), protein quantification, and reproducibility evaluation. Identified proteins received functional annotation and Gene Set Enrichment Analysis (GSEA), with subsequent differential expression analysis identifying significant differentially expressed proteins (DEPs). These DEPs were further analyzed through functional categorization, pathway enrichment examination, and expression pattern clustering.

### RNA Interference

Gene silencing was performed using PPARγ-specific siRNA and scrambled control (Suzhou Genepharma, China). Transient transfection was conducted in 6-well plates (5 × 10⁴ cells/well) using Lipofectamine 3000 (Thermo Fisher, USA) according to manufacturer’s protocol. Post-transfection incubation for 48 h preceded downstream analyses.

### Drug Treatment and Experimental Groups

To simulate the acute inflammatory phase of RA, LPS-induced inflammation model was established by 12 h exposure to 1 µg/mL LPS (Sigma-Aldrich, USA). Recombinant human METRNL (MedChemexpress; HY-P7065) was dissolved in sterile PBS (1 µg/mL stock). Two experimental paradigms were implemented. The first part was a dose-response study: 0 ng/mL, 50 ng/mL, 100 ng/mL, 200 ng/mL, and 300 ng/mL. The second part was a mechanism study investigated whether Metrnl exert its functions in RA-FLS through PPARγ: si-NC (negative control) group, Metrnl + si-NC group, Metrnl-group (control group), and Metrnl + PPARγ-siRNA group (experimental group). Different molecular biology techniques after 48 h-intervention were used to detect the experimental results.

### Apoptosis Analysis by Flow Cytometry

Cell suspensions were prepared using 0.25% trypsin-EDTA and washed twice with ice-cold PBS. After centrifugation (1000 rpm, 5 min), cells were resuspended in binding buffer at 1 × 10⁶ cells/mL. Staining performed with 5 µL Alexa Fluor 488-Annexin V and 10 µL PI (20 µg/mL; BD Pharmingen) prior to acquisition on BD FACSVerse™ analyzer.

### Cell Viability Assay

Cells seeded in 96-well plates (5 × 10³ cells/well) were treated as per experimental design. To prevent evaporation, added 100µL of PBS to the outer edge of each well. Cell Counting Kit (CCK)-8 reagent was added after 48 h treatment. Following 1 h incubation, absorbance was measured at 450 nm using Cytation5 reader (BioTek, USA; normalization wavelength 650 nm).

### Enzyme-Linked Immunosorbent Assay (Elisa)

Supernatants collected from RA-FLS cell cultures were aliquoted into 1.5 mL EP tubes. Quantitative analysis of inflammatory cytokines (IL-6, IL-17, TNF-α) and angiogenic factors (VEGF, PDGF) was performed using corresponding human ELISA kits (Beyotime Biotechnology, China) according to the manufacturer’s protocol. Briefly, capture antibody-coated plates underwent overnight incubation at 4 °C followed by three washes with PBS and thorough drying. Subsequently, 100 µL aliquots of cell supernatant were added to each well and incubated for 2 h at room temperature. After washing, detection antibodies were introduced for additional 2 h incubation. Following three subsequent washes, 100 µL Streptavidin-HRP conjugate was added for 20 min dark incubation. After final washing steps, 100 µL chromogenic substrate solution was incubated in darkness for 20 min prior to reaction termination with 50 µL stop solution. Optical density was immediately measured at 450 nm using a Cytation5 microplate reader (BioTek, USA), with sample concentrations determined through standard curve interpolation.

### Reverse Transcription-Polymerase Chain Reaction (RT-qPCR)

Total RNA was extracted from RA-FLS cells using TRIzol reagent (Thermo, USA). The RNA concentrations in each sample were measured by N60 Touch nanophotometer (Implen GmbH, Germany). Equal amounts of RNA (0.5 µg) were reverse transcribed into cDNA with commercial reagent (Thermo, USA). Subsequent real-time PCR was performed with qPCR Master Mix (YEASEN, China) on a Real-Time PCR system (Thermo, USA). The relative mRNA levels of target genes were calculated according to fold difference = 2^−ΔΔCt. Data were normalized against GAPDH expression. Primer sequences are provided in Table S1.

### Western Blot Analysis

Total protein extraction from RA-FLS cells was performed using RIPA lysis buffer (GenStar, China) and lysates were collected in 1.5 mL microcentrifuge tubes. Protein concentration was quantified via bicinchoninic acid (BCA) assay, with absorbance at 562 nm measured using a microplate reader (Molecular Devices, USA). Protein samples were resolved on 12% SDS-PAGE gels (GenStar, China) and transferred onto PVDF membranes (Merck, Germany) using a semi-dry electrophoretic transfer system at 20 V for 30 min. Membranes were blocked with 5% bovine serum albumin (BSA) for 2 h. Following this, membranes were overnight incubation at 4 °C with primary antibodies diluted in 5% BSA/TBST: rabbit monoclonal antibodies against human PPARγ: rabbit anti-homo PPARγ antibody (Abcam, UK), rabbit anti-homo IL-6 antibody (Abcam, UK), rabbit anti-homo IL-17 antibody (Abcam, UK), rabbit anti-homo TNF-α antibody (Abcam, UK), rabbit anti-homo VEGF antibody (Abcam, UK), rabbit anti-homo PDGF antibody (Abcam, UK), and rabbit anti-homo GAPDH antibody (Abcam, UK). After 1 h equilibration to room temperature, membranes were washed three times with TBST (10 min/wash) and incubated with IgG-HRP secondary antibody (1:1000; ThermoFisher, USA) for 2 h at room temperature. Following three additional washes, protein bands were visualized using enhanced chemiluminescence (ECL) substrate (Millipore, USA). Band intensities were quantified by densitometric analysis using ImageJ software (NIH, USA) and normalized to GAPDH expression.

### CIA Mouse Model Inducing Arthritis and Treatment Protocol

This research has received approval from the Experimental Animal Welfare Ethics Committee of China Medical University (CMU2023841). A total of 45 female DBA1J mice (high CIA susceptibility strain, 7 weeks, 18–23 g) were purchased from Jiangsu GemPharmatech Co., Ltd (China). After a week of adaptation in SPF animal center, mice were randomly classified into three groups (with 15 mice per group): the control group, the CIA model group, and the Metrnl treated CIA model group. All mice were fasted overnight before the primary immunization and booster immunization operations. Conduct the primary immunization on day 1, injecting 0.1mL emulsion mix of Complete Freund’s Adjuvant (Chondrex; catalog no. 7008) and Chick type II collagen (Chondrex; catalog no. 20012) on the site tail 2 cm. Conducted the booster immunization on Day 21, injecting 0.1mL emulsion mix of Incomplete Freund’s Adjuvant (Chondrex; catalog no. 7002) and collagen at a distance of 3 cm from the base of the tail. From Day 22, the Metrnl + CIA group administrated intraperitoneal injection Metrnl recombinant protein 100ng (1 µg/mL, 0.1mL, nearly 5 µg/kg) every three days for 41 days until Day 63. The CIA group was injected 0.1mL saline solution and the control group received no treatment. Observe the onset of arthritis since Day 22 and evaluate the arthritis by a qualitative clinical score (Table S2). Record the general state of the mice, including diet, behavior, and mood, while measuring their weight in each group weekly.

### Micro-CT Scanning and Bone Parameters Analysis

All mice were euthanized by overdose inhalation of Isoflurane (RWD; #R510-22-10) on Day 63 and subjected to pathological sampling forelimbs and hindlimbs. Bilateral forelimbs and hindlimbs were preserved in 4% paraformaldehyde (pH 7.4) at 4 °C prior to high-resolution micro-CT scanning using Skyscan 1276 system (Bruker, Belgium). All scans were performed by certified technicians at the Laboratory Animal Center of China Medical University under standardized protocols. The Micro-CT accompanying software NRecon (Bruker, Germany) was employed to convert the raw images acquired from Micro-CT into tomographic images. Three-dimensional rendering was performed using CTvox (v3.3.0) with volume of interest (VOI) defined as 2 mm distal to growth plate, while cross-sectional analysis utilized DataViewer (v1.5.6.2). Global thresholding was applied with grayscale range 95–255 for bone segmentation. Quantitative parameters included: total volume (TV, mm³), bone volume (BV, mm³), bone volume fraction (BV/TV, %), tissue surface (TS, mm²), bone surface (BS, mm²), bone surface/volume ratio (BS/BV, mm⁻¹), and bone surface density (BS/TV, mm⁻¹).

### Decalcification and Embedding

Forelimb and hindlimb specimens underwent decalcification in 10% ethylenediaminetetraacetic acid (EDTA) solution maintained at 4 °C for 45 ± 3 days, with solution replacement every 48 h. Decalcification progression was monitored through periodic needle-puncture testing until complete calcium dissolution was achieved. Coronal Sect. (5 μm thickness) were obtained in the anteroposterior plane using a rotary microtome (Leica RM2235, Germany).

### Histological Analysis

The specimen slices were dewaxed through the following steps: immersion in xylene I and II for 10 min each, followed by 5 min in isopropyl alcohol I, II, 90% ethanol, and 75% ethanol, and finally rinsed with distilled water. Hematoxylin-eosin (HE) staining (Sigma, Germany) and safranin O-fast green staining (Sigma, Germany) were performed according to standard protocols. The slides were scanned using the Converter Automatic Pathology Slide Scanner (3DHistech, Hungary), and CaseViewer software was employed for comprehensive analysis.

Tissue sections were sequentially dewaxed through xylene I/II (10 min each), followed by graded ethanol hydration (100% isopropanol I/II, 90% ethanol, 75% ethanol; 5 min each). After thorough distilled water rinsing, sections underwent hematoxylin-eosin (HE) and safranin O-fast green staining (Sigma-Aldrich, USA) following manufacturer-recommended protocols. Digital histopathological imaging was performed using the Converter Automatic Pathology Slide Scanner (3DHistech, Hungary). Quantitative histomorphometry analysis was conducted using CaseViewer 2.4 software (3DHistech) with standardized measurement protocols.

### Statistical Analysis

Data analysis was performed using SPSS software (IBM Corporation, Armonk, NY,580 USA), and figures were generated with OriginPro software (OriginLab Corporation,581 Northampton, MA, USA). Data adhering to normality were described using mean and standard deviation, and analyzed with two independent sample t-tests to compare. One-way ANOVA was employed for conducting group comparisons with a significance level being set as *p* < 0.05. Categorical data were analyzed by employing the Mann-Whitney U test and Fisher’s exact test to assess group differences and associations.

## Results

### Proteomic Analysis of Metrnl Stimulation on RA-FLS Cells

#### Metrnl Modulated RA-FLS Protein Expressions

To preliminarily explore the effect of Metrnl on human rheumatoid arthritis synovial cells and to support future research, total protein was extracted for quantitative protein sequencing. A total of 314 DEPs were identified (|log2FC| ≥ 1.5, *p* < 0.5), with 172 up-regulated and 142 down-regulated proteins (Figure S1A). Key up-regulated proteins included TNFRSF1A, IFI44, MX2, VASN, ZNF428, MPP2, PARP14, PPP1R10, SLC4A2, and UBL5, while key down-regulated proteins included STC1, SCAF1, SEMA3C, CEMIP, SORT1, CCBE1, TPM1, MYL9, PRG4, and DLC1.

#### GSEA Reveals Metrnl’s Multifaceted Role in Angiogenesis Suppression and Immune-Inflammatory Network Activation

**Fig. 1:**
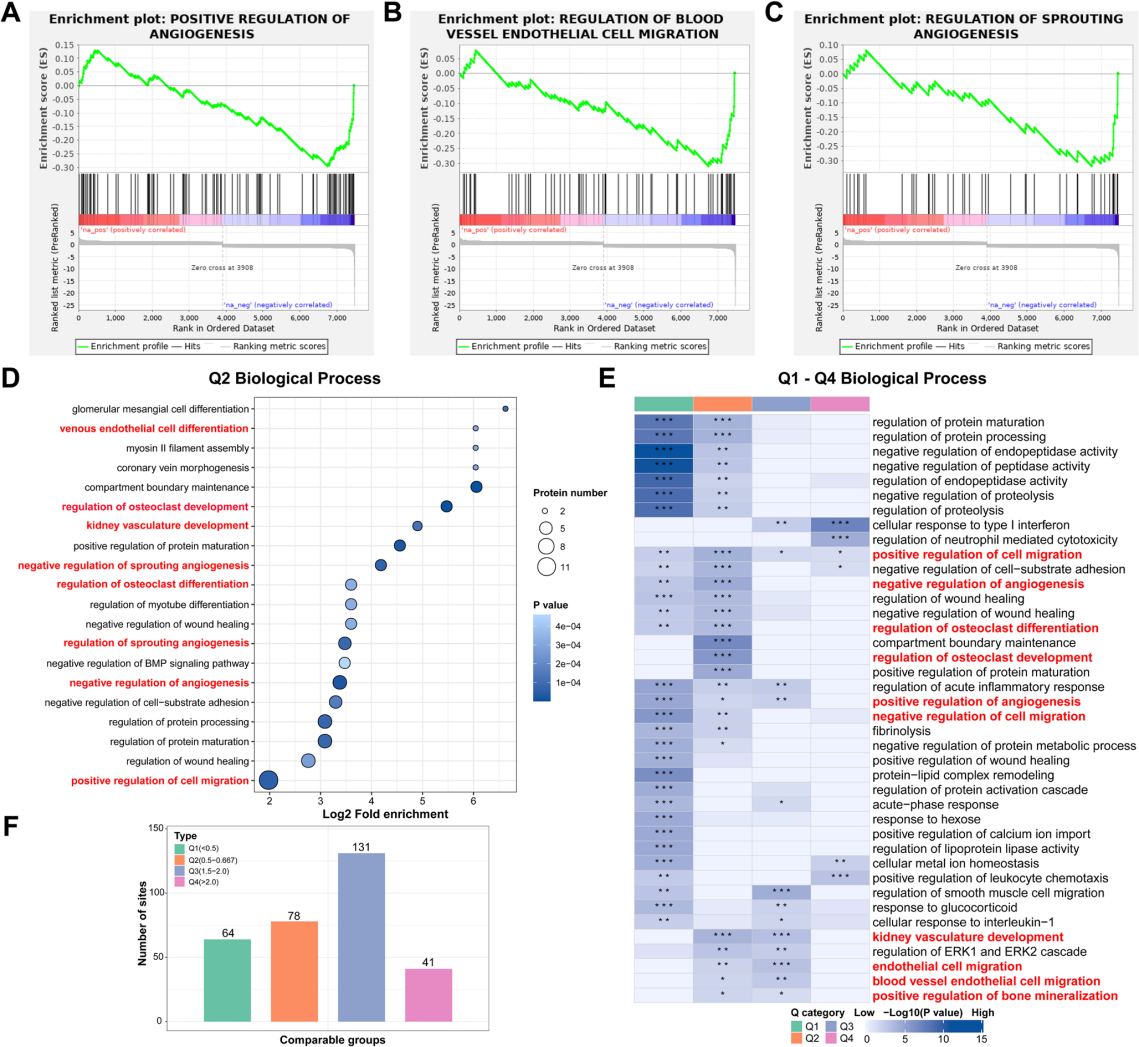
Proteomic Analysis of Metrnl Stimulation on RA-FLS cells. (**A**) GSEA map of negatively regulated Positive Regulation of Angiogenesis. (**B**) GSEA map of negatively regulated Regulation of Blood Vessel Endothelial Cell Migration. (**C**) GSEA map of negatively regulated Regulation of Sprouting Angiogenesis. (**D**) GO enrichment map in the biological process category of Q2 cluster category DEPs. (**E**) GO enrichment map in the biological process category of total Q1 - Q4 cluster category DEPs. (**F**) Q1-Q4 categories of different fold enrichment DEPs

GSEA based on the Gene Ontology (GO) database revealed significant downregulation of angiogenesis-associated biological processes, particularly in three key pathways: Positive Regulation of Angiogenesis (Fig. 1A, *p* < 0.001), Regulation of Blood Vessel Endothelial Cell Migration (Fig. 1B, *p* = 0.002), and Regulation of Sprouting Angiogenesis (Fig. 1C, *p* = 0.026). These findings strongly suggest that Metrnl may function as a pivotal regulator in modulating both angiogenic processes and endothelial cell motility.

Through KEGG pathway analysis, GSEA demonstrated coordinated upregulation of pathways involved in inflammatory immune responses and vascular biology (NES > 0; all *p* < 0.05). Notably enhanced pathways included TNF Signaling Pathway (Figure S1B, *p* < 0.001), Cytokine-Cytokine Receptor Interaction (Figure S1C, *p* < 0.001), T Cell Receptor Signaling Pathway (Figure S1D, *p* = 0.009), Toll-like Receptor Signaling Pathway (Figure S1E, *p* = 0.002), Th17 Cell Differentiation (Figure S1F, *p* = 0.018), and VEGF Signaling Pathway (Figure S1G, *p* = 0.042). This concerted activation pattern implies Metrnl’s promotive role in establishing an immune-inflammatory-angiogenic network, potentially creating a microenvironment conducive to infection response and autoimmune pathogenesis. Of particular interest, we observed downregulation enrichment in Complement and Coagulation Cascades (Figure S1H, *p* < 0.001) and Rheumatoid Arthritis-related pathways (Figure S1I, *p* = 0.032), indicating Metrnl’s multifaceted involvement in both innate immune regulation and potential modulation of hemostatic processes.

#### Clustering Analysis Reveals Metrnl’s Impact on Coordinates Multidirectional Regulation of Vascular Homeostasis

DEPs were categorized into four clusters (Q1–Q4) based on fold enrichment in GO database (Fig. 1F). Functional clustering analysis was performed and focused on pathway proteins related to the RA pathogenesis (Fig. 1E). Especially, Q2 cluster analysis revealed Metrnl’s multidirectional impact on coordinating vascular homeostasis, where balancing angiogenesis, osteoclast genesis, and renal vasculature development (Fig. 1D).

### Metrnl Promotes PPARγ Expression in LPS-induced RA-FLS Cells

**Fig. 2:**
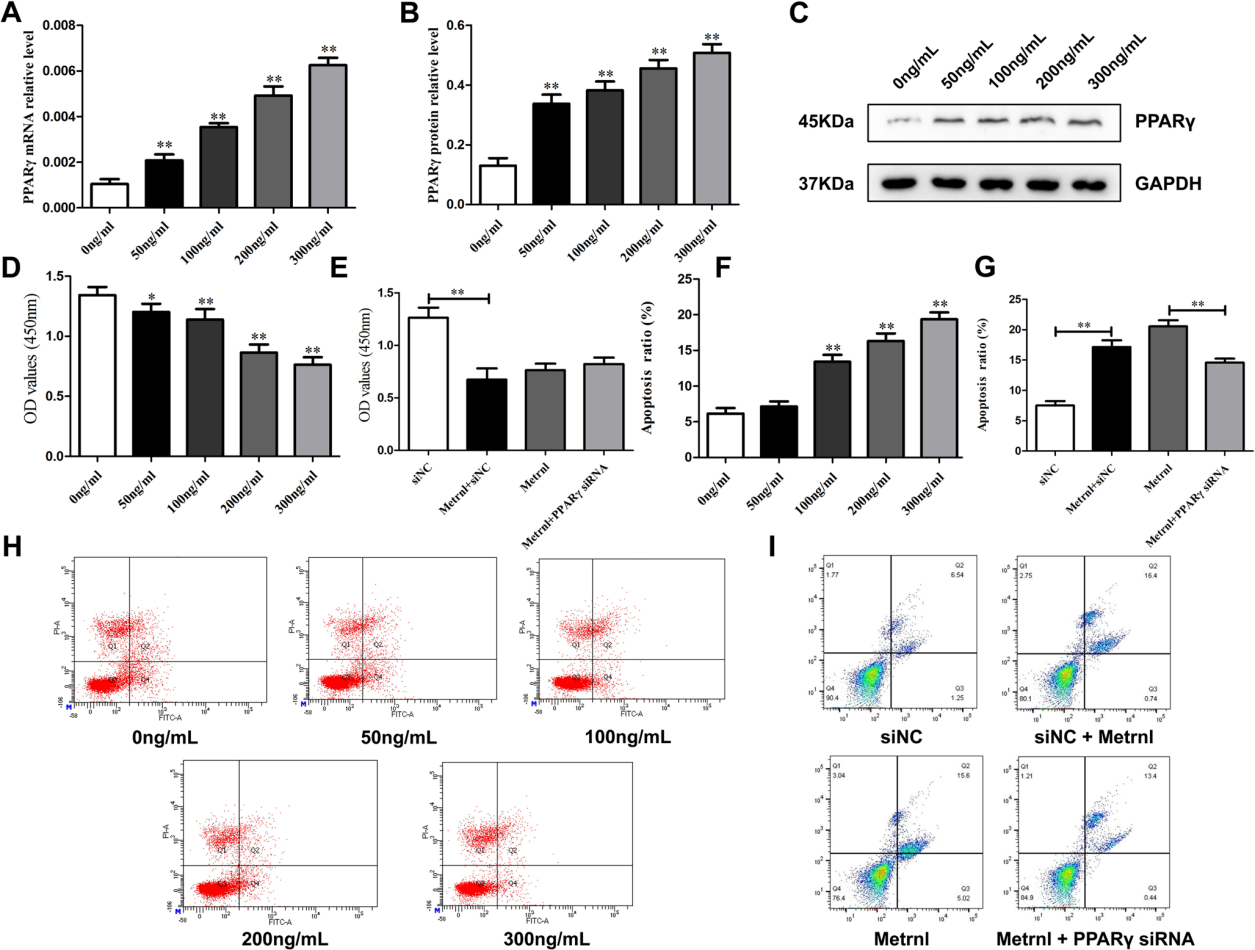
Metrnl promotes apoptosis in LPS-induced RA-FLS cells and inhibits their proliferation by regulating PPARγ expression. (**A**) The mRNA level of PPARγ expression in Metrnl-treated LPS-induced RA-FLS cells. (**B-C)** WB analysis of PPARγ in Metrnl-treated LPS-induced RA-FLS cells. (**D**) Metrnl treatment on the cell proliferation of LPS-induced RA-FLS cells. (**E**) Metrnl treatment on cell proliferation by blocking PPARγ using siRNA in RA-FLS cells. (**F**) Flow cytometry detected Metrnl treatment on the apoptosis rate in LPS-induced RA-FLS cells. (**G**) Flow cytometry detected Metrnl treatment on the apoptosis rate by blocking PPARγ using siRNA in LPS-induced RA-FLS cells. (**H**) Representative graphs showed the effects of Metrnl treatment on cell apoptosis in LPS-induced RA-FLS by flow cytometry. (**I**) Representative graphs showed the effects of Metrnl treatment on cell apoptosis by blocking PPARγ by siRNA in LPS-induced RA-FLS by flow cytometry.

To simulate an inflammatory environment in synovium cells, we stimulated RA-FLS cells with 1 µg/mL LPS to develop an inflammatory cell model. We then applied different concentrations of Metrnl recombinant protein (0 ng/mL, 50 ng/mL, 100 ng/mL, 200 ng/mL, and 300 ng/mL) to LPS-induced RA-FLS cells to investigate the direct effects of Metrnl stimulation on PPARγ expression. Results showed Metrnl significantly increased PPARγ mRNA (Fig. 2A) and protein (Fig. 2B-C) expression levels compared to the control, also exhibiting a positive correlation with Metrnl concentration. This indicated that Metrnl could enhance PPARγ expression in RA-FLS cells; subsequent studies will examine whether it modulates cell proliferation and apoptosis via PPARγ.

### Metrnl Suppresses LPS-induced RA-FLS Cells Proliferation by Regulating PPARγ Expression

To clarify the effects of Metrnl on cell proliferation in LPS-induced RA-FLS cells, CCK8 assays were conducted to assess cell viability. The control group received no treatment (0 ng/mL), while the experimental groups received treatments with 50 ng/mL, 100 ng/mL, 200 ng/mL, and 300 ng/mL of Metrnl. After 48 h of culture, the OD value in the Metrnl-treated group showed a significant decrease compared with the control group in a concentration-dependent way, indicating that Metrnl inhibits RA-FLS cells proliferation (Fig. 2D). Then PPARγ-siRNA was employed to targeted silence PPARγ expression and evaluate subsequent changes. The experiment was divided into four groups: si-NC (negative control) group, Metrnl + si-NC group, Metrnl-group (control group), and Metrnl + PPARγ-siRNA group (experimental group). All experimental groups receiving Metrnl were given a concentration of 300 ng/mL. The OD value in the Metrnl + si-NC group decreased markedly than that in the si-NC group (Fig. 2E). Although the proliferation in Metrnl + PPARγ-siRNA group was higher than that observed in the Metrnl-group, this difference did not reach statistical significance. These results suggest that Metrnl inhibits RA-FLS cell proliferation by regulating PPARγ expression.

### Metrnl Promotes LPS-induced RA-FLS Cells Apoptosis by Regulating PPARγ Expression

Similar to the previous section, we further examined the effects of Metrnl on cell apoptosis in LPS-induced RA-FLS cells. Flow cytometry analysis revealeda marked rise on apoptosis in the Metrnl treatment groups compared with the control, except for the 50 ng/mL concentration (Fig. 2F and H). This suggested that Metrnl could promote RA-FLS cells apoptosis at concentrations exceeding 100 ng/mL. Following the employment of PPARγ-siRNA, the apoptosis rate in the Metrnl + si-NC group was remarkably elevated compared to the si-NC group (Fig. 2G and I). Additionally, relative to the Metrnl-group, RA-FLS cell apoptosis in the Metrnl + PPARγ-siRNA group was significantly reduced. These findings suggest that Metrnl can promote LPS-induced RA-FLS cell apoptosis, while PPARγ may effectively target and silence its effects.

3.5 Metrnl inhibits the secretion and expression of inflammatory cytokines and angiogenic factors in LPS-induced RA-FLS cells.

To investigate the effects of direct Metrnl stimulation on cytokines and angiogenic factors secretion and expression in LPS-induced RA-FLS cells, we assessed the secretion levels of IL-17, IL-6, and TNF-α in cell supernatants by Elisa assays as well as their intracellular mRNA by RT-qPCR and protein contents by western blot. The experimental groups were treated with 50 ng/mL, 100 ng/mL, 200 ng/mL, and 300 ng/mL of Metrnl, while the control group received no treatment.

**Fig. 3:**
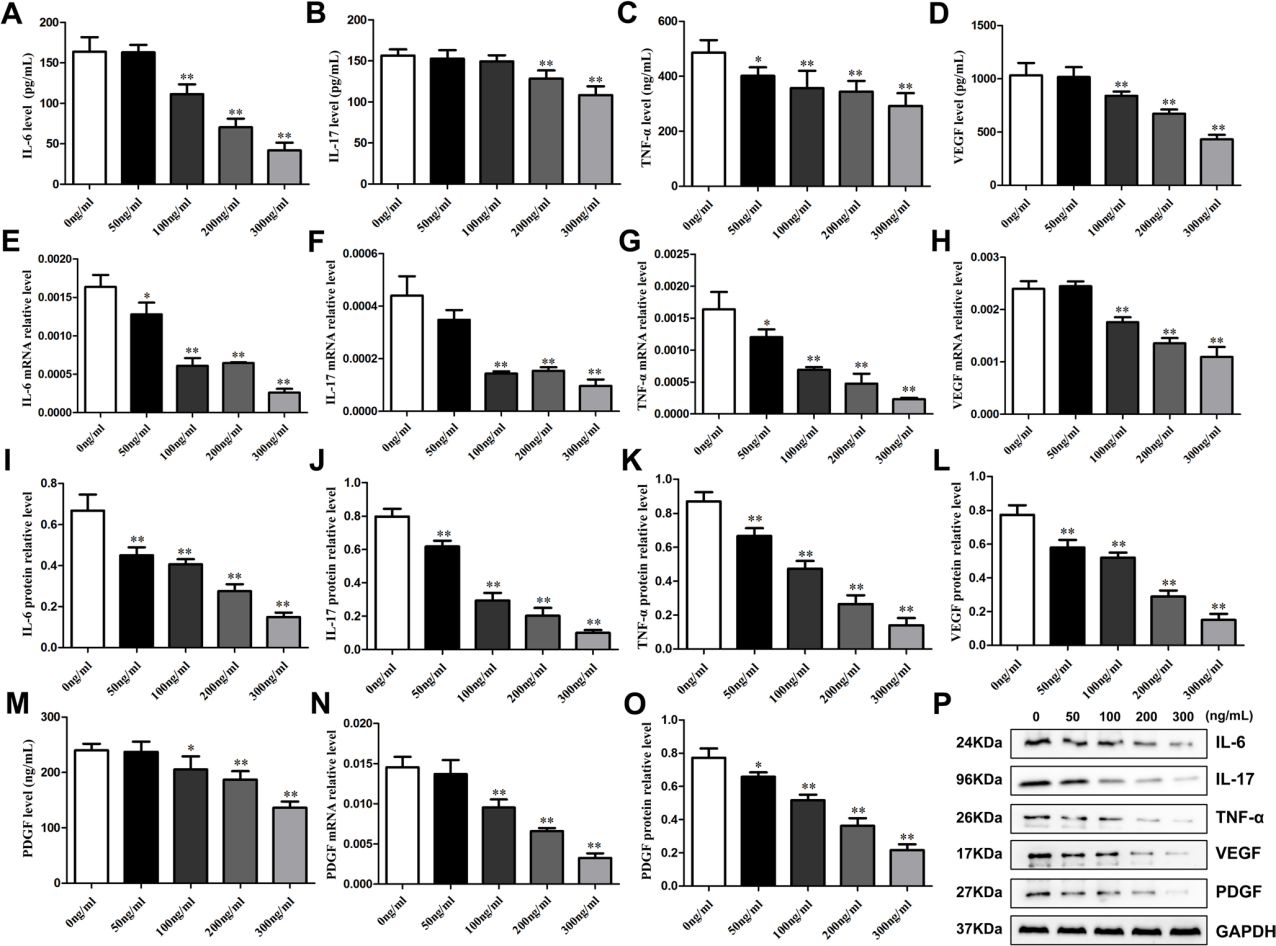
Effects of Metrnl intervention on cytokine and angiogenesis factor secretions in LPS-induced RA-FLS cells. (**A-D**) Elisa assays measured concentrations of IL-6, IL-17, TNF-α, and VEGF in cell supernatant upon Metrnl intervention. (**E-H**) The mRNA levels of IL-6, IL-17, TNF-α, and VEGF expressions in LPS-induced RA-FLS cells. (**I-L**) Quantitative protein analysis of IL-6, IL-17, TNF-α, VEGF expressions. (**M**) PDGF level in the cell supernatant. (**N**) The mRNA level of PDGF expression in LPS-induced RA-FLS cells. (**O**) Quantitative protein analysis of PDGF expression. (**P**) WB analysis of IL-6, IL-17, TNF-α, VEGF, and PDGF after Metrnl intervention in RA-FLS cells. *p < 0.05, **p < 0.01, ***p < 0.001

Regarding inflammatory cytokines, Metrnl resulted a general downward trend, but there were differences in specific changes. For the TNF-α, Metrnl resulted a pronounced decreasing trend compared to the control group, with the most significant inhibitory effect on secretion at 300 ng/mL (Fig. 3C and G, and 3K). For the IL-6, Metrnl significantly inhibited its mRNA and protein expression at 50 ng/mL, with the most pronounced effect at 300 ng/mL (Fig. 3E and I). However, IL-6 levels in the cell supernatants indicated that Metrnl exhibited inhibitory effects only at concentrations of 100 ng/mL or higher (Fig. 3A). For the IL-17, Metrnl significantly inhibit its mRNA expression at 100 ng/mL, with the inhibitory effect remaining unchanged with increased concentration (Fig. 3F). Besides, Metrnl only suppressed the secretion level of IL-17 in cell supernatants at concentrations of 200 ng/mL or higher (Fig. 3B). Notably, IL-17 protein levels were significantly reduced in a concentration-dependent manner upon Metrnl stimulation (Fig. 3J).

Regarding angiogenic factors, Metrnl significantly inhibited VEGF and PDGF secretion and expression. At the protein level, Metenl reduced both VEGF (Fig. 3L) and PDGF (Fig. 3O) protein expression in a concentration-dependent manner compared to control groups. At the mRNA level, Metrnl suppressed VEGF (Fig. 3H) and PDGF (Fig. 3N) mRNA transcription at concentrations of 100 ng/mL or higher. Furthermore, at the secretion level, Metrnl diminished the levels of VEGF (Fig. 3D) and PDGF (Fig. 3M) in cell supernatants when treated at 100 ng/mL or higher. These results suggested that Metrnl markedly inhibits both inflammatory cytokines and angiogenic factors secretion and expression in LPS-induced RA-FLS cells.

### Metrnl Reduces Inflammatory Cytokines and Angiogenic Factors Via PPARγ in RA-FLS Cells

To investigate the effects of Metrnl on the expression of inflammatory cytokines and angiogenesis-related factors in LPS-induced RA-FLS cells following PPARγ-siRNA-mediated silencing. The experimental groups remained consistent with previous part.

**Fig. 4:**
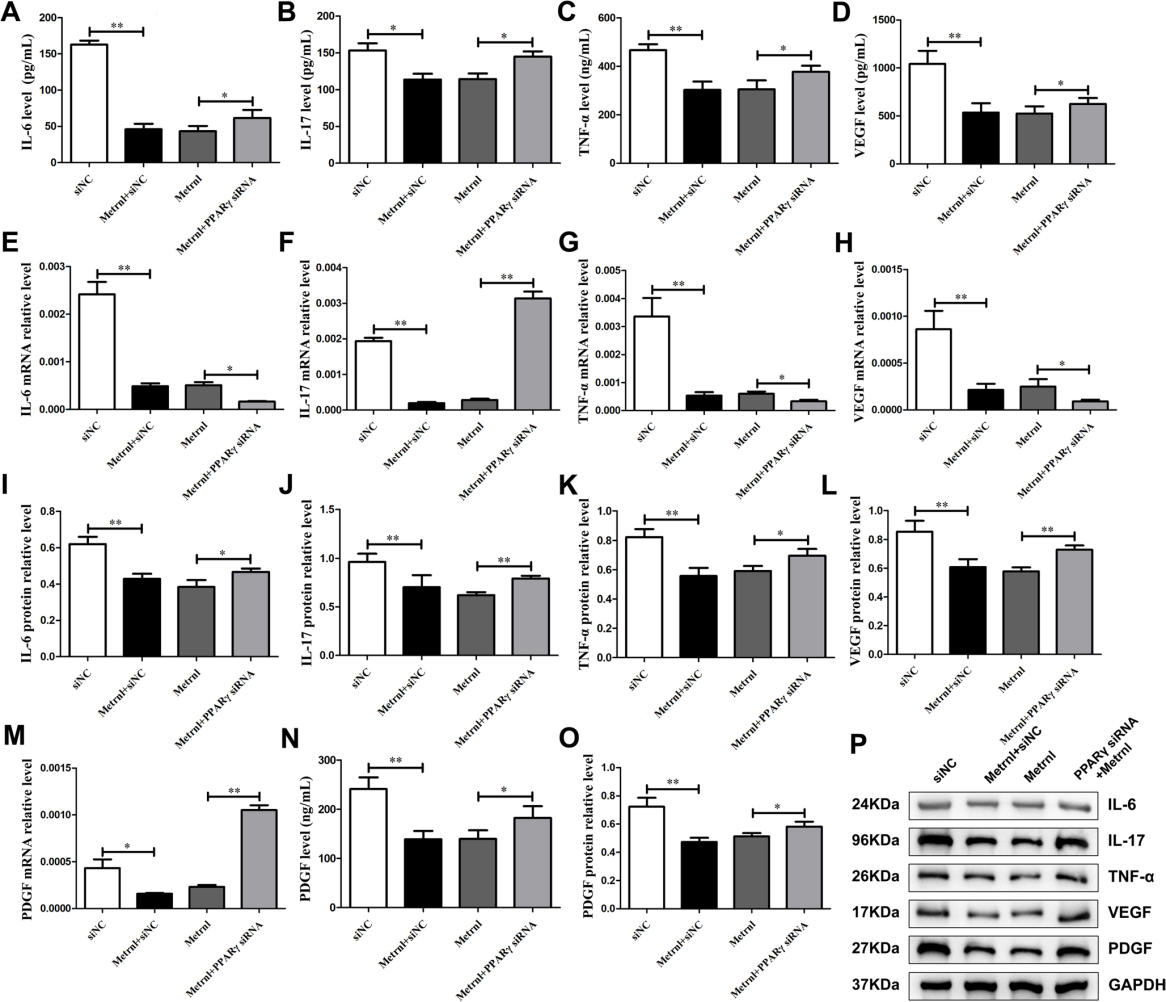
Effects of PPARγ blockage on Metrnl intervention in LPS-induced RA-FLS cells cytokine and angiogenesis factor secretions. (**A-D**) Blockade PPARγ on IL-6, IL-17, TNF-α, and VEGF secretion levels in the cell supernatant. (**E-H**) Blockade PPARγ on mRNA levels of IL-6, IL-17, TNF-α, and VEGF expressions in LPS-induced RA-FLS cells. (**I-L**) Blockade PPARγ on protein levels of IL-6, IL-17, TNF-α, VEGF expressions. (**M**) Blockade PPARγ on PDGF level in the cell supernatant. (**N**) Blockade PPARγ on PDGF mRNA level in LPS-induced RA-FLS cells. (**O**) Blockade PPARγ on PDGF protein level in LPS-induced RA-FLS cells. (**P**) Blockade PPARγ on WB analysis of IL-6, IL-17, TNF-α, VEGF, and PDGF after Metrnl intervention in RA-FLS cells. *p < 0.05, **p < 0.01, ***p < 0.001.

As shown in Fig. 4, compared with the si-NC group, the secretion, mRNA, and protein levels of IL-6, IL-17, TNF-α, VEGF, and PDGF in the Metrnl + si-NC group were significantly reduced. These results indicated Metrnl markedly suppresses the production of cytokines and angiogenic factors. In contrast, when compared to the Metrnl group, expression levels of IL-6, IL-17, TNF-α, VEGF, and PDGF were significantly increased in the Metrnl + PPARγ-siRNA group at both protein and secretion levels. Notably, at the mRNA level, IL-17 and PDGF expression levels were elevated in the Metrnl + PPARγ-siRNA group, suggesting that PPARγ-targeted silencing counteracted Metrnl’s inhibitory effects on IL-17 and PDGF mRNA expression. Conversely, the mRNA levels of IL-6, TNF-α, and VEGF remained decreased in the Metrnl + PPARγ siRNA group. These indicated that PPARγ-targeted silencing was ineffective in counteracting Metrnl’s inhibitory effects on IL-6, TNF-α, and VEGF mRNA expression.

Overall, Metrnl may serve as a protective mediator in the inflammatory environment of synovial cells in RA, inhibiting the inflammatory cytokine storm and angiogenesis. This protective role may through the regulation of PPARγ, which is manifested by a reduction in inflammatory cytokines and angiogenic factors.

### Metrnl Treatment Improves the Overall Health and Arthritis Progression in CIA Arthritis Model

After the booster immunization on Day 21, most mice exhibited signs of poor mental state and reduced appetite. From Day 23, both the CIA and Metrnl + CIA groups began to show arthritis symptoms, characterized by swelling in both forelimbs and hindlimbs. All mice in the CIA and Metrnl + CIA groups exhibited arthritis symptom onset by Day 30. However, no significant differences were observed in the timing of symptom onset between the two groups. Notably, nearly all mice experienced a rapid onset of arthritis that peaked in inflammation overnight with joint redness and swelling, rather than following a gradual chronic progression.

**Fig. 5:**
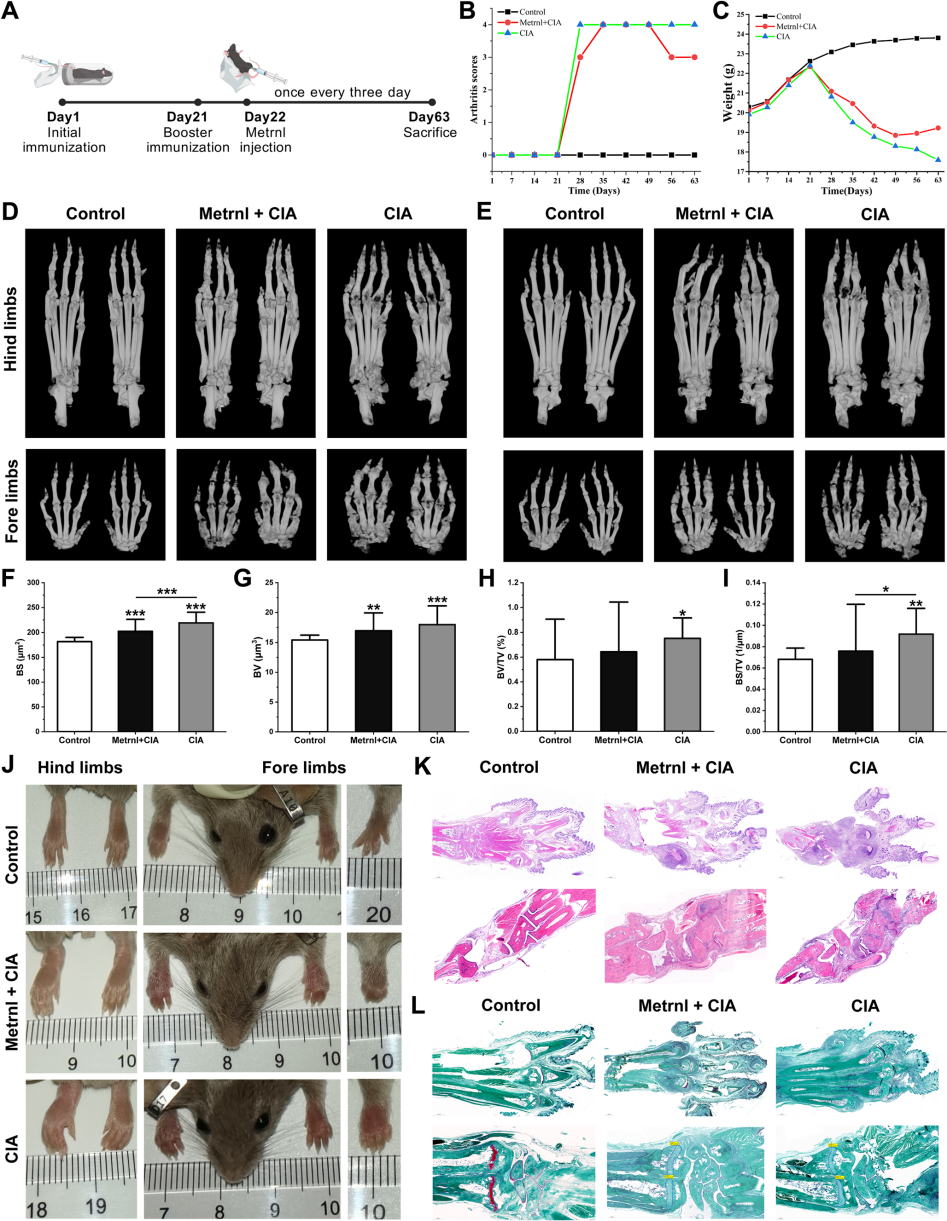
Metrnl treatment in RA arthritis mice model and histological analysis of fore and hind limbs. (**A**) The timeline for the construction procedure of RA mouse model and treatment regimen. (**B**) The scatter line chart of arthritis scores changes in mice over time. (**C**) The scatter line chart of mice weight changes over time. (**D-E**) The Micro-CT reconstruction images of the palmar/dorsal side of the mice fore/hind limbs. (**F-I**) The bone-related parameters with statistical differences of BS, BV, BV/TV, and BS/TV of mice. (**J**) The camera images of the mice fore and hind limbs after different treatments. (**K**) HE staining of ankle joints and palms to assess inflammation infiltration and synovial hyperplasia. (**L**) Safranin O-Fast staining of ankle joints and palms to evaluate cartilage destruction. *p < 0.05, **p < 0.01, ***p < 0.001

Regarding the general state, starting from Day 22, all onset mice showed a significant reduction in body weight and decreased activity levels markedly compared to healthy controls (Fig. 5B). On Day 63, the CIA group still exhibited severe swelling, with no obvious remission in inflammation. Besides, there was no increase in body weight, and joint deformities were noted alongside peripheral skin erythema. In contrast, the Metrnl group showed a slight decrease in both redness and swelling after one month of Metrnl treatment; however, some degree of swelling persisted on Day 63 (despite reduced skin erythema and inflammation), while body weight trended upward. Notably, the CIA + Metrnl group maintained a higher body weight than the CIA group, suggesting that Metrnl treatment may improve the mental state of CIA mice and reduce disease-related losses.

Interestingly, there was no significant difference in the degree of joint swelling between the CIA and Metrnl + CIA groups during the onset stage, with both exhibiting severe swelling (Fig. 5C). However, a notable difference was observed in the number of swollen joints. In the CIA group, most mice displayed swelling in multiple limbs—either both forelimbs and hindlimbs or all four limbs—while instances of isolated limb swelling were exceedingly rare. In contrast, Metrnl + CIA group had fewer cases of simultaneous swelling across all four joints, with most cases showing severe swelling limited to either the single forelimbs or hindlimbs. Simultaneously, the swollen joints were characterized by pronounced symptoms of redness, swelling, heat, and pain, which clearly delineated them from the unaffected joints. These results indicated that Metrnl alleviated the arthritis progression and showed therapeutic effects in CIA arthritis model.

### Metrnl Attenuates Cartilage and Bone Destruction in CIA Arthritis Model

To evaluate the bone erosion and damage severity, we conducted the Micro-CT reconstructing and analysis. We separately reconstructed the palmar and dorsal surfaces of the forelimbs and hindlimbs (Fig. 5D and E). Compared to the control group, both the CIA group and Metrnl + CIA group showed apparent bone erosion and osteophyte formation. However, the bone destruction in the CIA group was more severe, accompanied by bone and cartilage erosion. Besides, on Day 63, some CIA mice showed joint deformities and degeneration. We further analyzed the bone parameters of Micro-CT. Results showed that the BS of CIA mice was significantly higher than the Metrnl + CIA group, with both groups exhibiting marked increases relative to the control group, suggesting that Metrnl reduce area expansion of bone destruction (Fig. 5F). Besides, indicators including BV (Fig. 5G), BV/TV (Fig. 5H), and BS/TV (Fig. 5I) all showed significant elevated in CIA group compared to Metrnl + CIA group, while no differences in TS (Figure S2A), BS/BV (Figure S2B), and TV (Figure S2C). These increased indicators suggest that bone synthesis is greater than bone resorption, leading to an increase in bone volume. Given that our arthritis model was conducted at the acute inflammatory outbreak stage, a greater increase in bone synthesis indicates more severe bone damage, necessitating the formation of additional new bone to replace the eroded bone tissue.

HE staining of joint tissue sections was performed to evaluate joint pathology in arthritis mice (Fig. 5K, Figure S3A). In the control group, the joint surface was intact, exhibiting no inflammatory cell infiltration or proliferation within the synovial fluid, and cells were organized in a regular manner. In contrast, the CIA group displayed pronounced hyperplasia of the synovial membrane, characterized by disorganized and loosely arranged cells. A substantial accumulation of inflammatory cells was observed, alongside varying degrees of erosion and damage to both cartilage and bone tissue. While in the CIA + Metrnl group, the joint surface remained relatively intact, demonstrating reduced synovial hyperplasia along with decreased inflammatory cell infiltration. These suggested that Metrnl could alleviate inflammatory cell infiltration, joint synovial hyperplasia, and bone destruction in arthritis mouse models.

Since severe cartilage destruction can occur near hyperplastic synovial vascular pannus in RA, we used the safranin-O-fast green staining method to evaluate Metrnl’s effect on cartilage damage in the ankle joints (Fig. 5L, Figure S3B). Regarding the control group, well-defined deep red transparent cartilage was observed. In contrast, both the CIA group and the CIA + Metrnl group showed blurred cartilage staining, indicating varying degrees of cartilage damage. Notably, compared to the CIA group, the Metrnl-treated group demonstrated more uniform red staining of the cartilage matrix and a more regular arrangement of chondrocytes, with their numbers approaching those in the control. Furthermore, cartilage damage in the CIA + Metrnl group was significantly reduced. These findings suggested that Metrnl may exert a therapeutic effect on cartilage damage in CIA arthritis mice.

## Discussions

In the acute active period of RA, a substantial accumulation of inflammatory cells is observed in the synovial fluid, contributing to joint deformities and serving as a significant risk factor for adverse prognoses [20]. Fibroblast-like synoviocytes (FLS) represent the predominant cell population within the synovial tissue, where their excessive proliferation and insufficient apoptosis are the pathological basis of RA [21]. Studies have shown that RA-FLS and the surrounding synovial fluid of RA exhibit elevated levels of inflammatory cytokines such as IL-6, IL-17, and TNF-α [22]. Moreover, these cytokine levels correlate positively with the extent of joint damage associated with RA. Consequently, elucidating both the underlying mechanisms driving disease progression and effective treatment strategies for RA is imperative; understanding how these inflammatory cytokines are expressed will be pivotal in developing novel targeted therapies.

Angiogenesis, the sprouting of new capillaries from pre-existing vessels, is essential for development and tissue repair, yet it also fuels autoimmune and inflammatory diseases such as RA [23]. Based on our proteomic profiling results, we identified Metrnl as a pivotal regulator of endothelial migration, angiogenesis, and inflammation. Recent studies established Metrnl’s pro-angiogenic capacity in vitro [24–26], we observed context-dependent anti-angiogenic effects in RA pathogenesis, potentially mediated through PPARγ signaling. Accumulating evidence shows that Metrnl’s vascular effects are highly context-dependent. In low-inflammation settings of full-thickness skin wounds or myocardial ischemia, Metrnl can partner with macrophages to increase VEGF release and accelerate normal revascularization [27, 28]. By contrast, Metrnl administration in highly inflammatory settings, such as in a laser-induced choroidal neovascularization model, suppresses pathological neovascular formation [24], mirroring the inhibitory effects we observe in LPS-stimulated RA-FLS and CIA joints. Thus, the vascular effects of Metrnl depend more on the surrounding microenvironment than on the molecule itself. Collectively, Metrnl may act as a modulating factor during angiogenesis, promoting vessel growth during tissue repair while curbing aberrant angiogenesis in inflammatory environments.

Mechanistically, the nuclear receptor PPARγ, previously linked to Metrnl’s insulin-sensitizing effects in diabetes, appears central to this switch. In synovial fibroblasts, Metrnl down-regulates key inflammatory cytokines (IL-6, TNF-α, IL-17) and angiogenic mediators (PDGF, VEGF), thereby disrupting the self-amplifying inflammatory loop of RA that involves IL-6-mediated immune activation, TNF-α-induced synovial destruction, and IL-17-driven osteoclastogenesis [19, 29–33]. Notably, our LPS-stimulated RA-FLS model recapitulates early RA hallmarks including synovial hyperplasia and pathological angiogenesis, with Metrnl’s simultaneous targeting of FLS proliferation and inflammatory factor secretion underscoring its therapeutic potential. While contrasting with reported pro-angiogenic effects in choroidal neovascularization models, these findings highlight Metrnl’s microenvironment-dependent regulatory behavior that promoting vascular homeostasis under physiological conditions yet constraining pathological neovascularization in inflammatory settings. This dichotomy emphasizes the need for tissue-specific investigation of Metrnl’s mechanisms, particularly its interplay with VEGF signaling in balancing physiological endothelial migration versus synovial vascular inflammation.

The CIA mouse model is a mature animal model of RA, which extensively utilized to investigate the underlying mechanisms [34]. In this model, the activation of numerous inflammatory cytokines and angiogenic factors induces FLS-cells abnormal proliferation, leading to significant inflammatory cell accumulation within the joint synovium, vascular pannus formation, and ultimately resulting in destruction of both cartilage and bone tissue. Our experimental findings demonstrated that Metrnl effectively inhibited the progression of cartilage and bone degradation in CIA mice. Pathological staining revealed a marked reduction in cartilage damage within the joint tissues upon Metrnl treating. Additionally, Metrnl suppressed inflammatory cells infiltration at the joint periphery as well as vascular pannus formation, thereby mitigating bone destruction. These results provide additional evidence that Metrnl protects CIA mice from joint-bone degradation. Nonetheless, our study has several limitations. On the one hand, the in-vivo experiments were conducted with a single Metrnl dose (100 ng/0.1 mL, almost nearly 5 µg/kg), chosen based on a previous report [18], while a comprehensive dose-response analysis has yet to be performed. Future work should therefore define the optimal therapeutic window and establish a safe dosing range. On the other hand, although we have evaluated the effects of Metrnl on inflammatory cytokine expression at the cellular level, we did not perform immunohistochemical staining to quantify specific inflammatory cell subsets in CIA joint sections. As a result, our study lacks quantitative data on the extent of inflammatory cell infiltration.Taken together, our results showed that Metrnl inhibit the proliferation of LPS-induced RA-FLS cells and promote their apoptosis, while downregulating the expression of inflammatory and angiogenic factors via regulating PPARγ. Besides, Metrnl attenuated cartilage and bone destruction, exerted a therapeutic effect on cartilage and bone damage in CIA arthritis mice model. In conclusion, our study reports for the first time that Metrnl exhibits strong anti-inflammatory and anti-angiogenic effects in RA synovial cells, potentially through METRNL-mediated PPARγ signaling. These results may reveal new insights into the pathological mechanisms of RA and suggest a novel therapeutic strategy to inhibit its onset and progression.

## Supplementary Information

Below is the link to the electronic supplementary material.

Supplementary Material 1

## Data Availability

No datasets were generated or analysed during the current study.
