# Supplementary material for: Metrnl/Meteorin-like/IL-41 Alleviates Rheumatoid Arthritis Via PPARγ-Mediated Suppression of Inflammation, Angiogenesis, and Bone Destruction
Source: Inflammation. 2026 Jan 6;49(1):31. doi: 10.1007/s10753-025-02426-x (PMC12832592; doi:10.1007/s10753-025-02426-x)
Supplement: Supplementary file 1 — Supplementary Material 1 [file 10753_2025_2426_MOESM1_ESM.docx]

Table S1. Primer sequences for the mRNA of the target genes

| **Gene** | **Primer sequences** | **Length** |
| --- | --- | --- |
| IL-6 | Forward: 5'-TGAGGAGACTTGCCTGGT -3'  Reverse: 5'-GGGTCAGGGGTGGTTATT -3' | 186bp |
| IL-17 | Forward: 5'-TACAACCGATCCACCTCAC-3'  Reverse: 5'-GTAGTCCACGTTCCCATCA-3' | 126bp |
| TNF-α | Forward: 5'-CCCTCCTTCAGACACCCT -3'  Reverse: 5'-GGTTGCCAGCACTTCACT -3' | 153 bp |
| VEGF | Forward: 5'-CAGGGAAGAGGAGGAGATG-3'  Reverse: 5'-CTGGGTTTGTCGGTGTTC -3' | 148bp |
| PDGF | Forward: 5'-CTGATGAAAGCACACGGA -3'  Reverse: 5'-TGATCTCTGGATGTCGGAA-3' | 143bp |
| GAPDH | Forward: 5'-CAAGTTCAACGGCACAGTCAAG-3’  Reverse: 5'-ACATACTCAGCACCAGCATCAC-3’ | 123 bp |

Table S2. Qualitative arthritis scoring.

| **Score** | **Condition** |
| --- | --- |
| 0 | Normal |
| 1 | Mild, but definite redness and swelling of the ankle or wrist, or apparent redness and swelling limited to individual digits, regardless of the number of affected digits |
| 2 | Moderate redness and swelling of ankle or wrist |
| 3 | Severe redness and swelling of the entire paw including digits |
| 4 | Maximally inflamed limb with involvement of multiple joints |





Figure S1. Proteomics analysis of Metrnl stimulation on RA-FLS cells.

(A) DEPs of comparable group between Metrnl and control group. (B) GSEA map of positively regulated TNF Signaling Pathway. (C) GSEA map of positively regulated Cytokine-Cytokine Receptor Interaction. (D) GSEA map of positively regulated T Cell Receptor Signaling Pathway. (E) GSEA map of positively regulated Toll-like Receptor Signaling Pathway. (F) GSEA map of positively regulated Th17 Cell Differentiation. (G) GSEA map of positively regulated VEGF Signaling Pathway. (H) GSEA map of negatively regulated Complement and Coagulation Cascades. (I) GSEA map of negatively regulated Rheumatoid Arthritis.


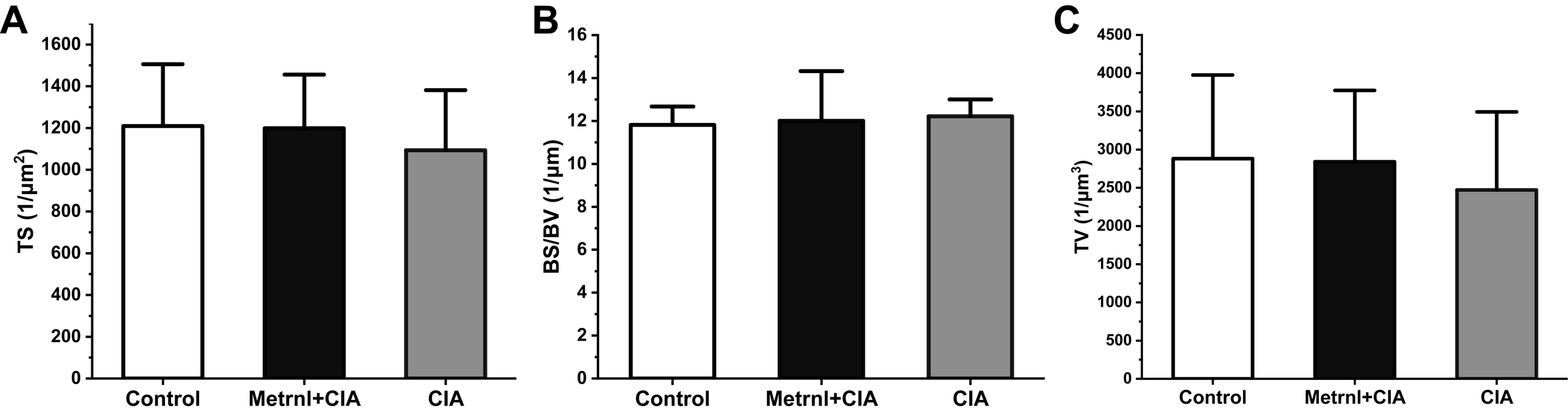


Figure S2. The bone-related parameters without statistical differences of TS, BS/BV, and TV in CIA mice.


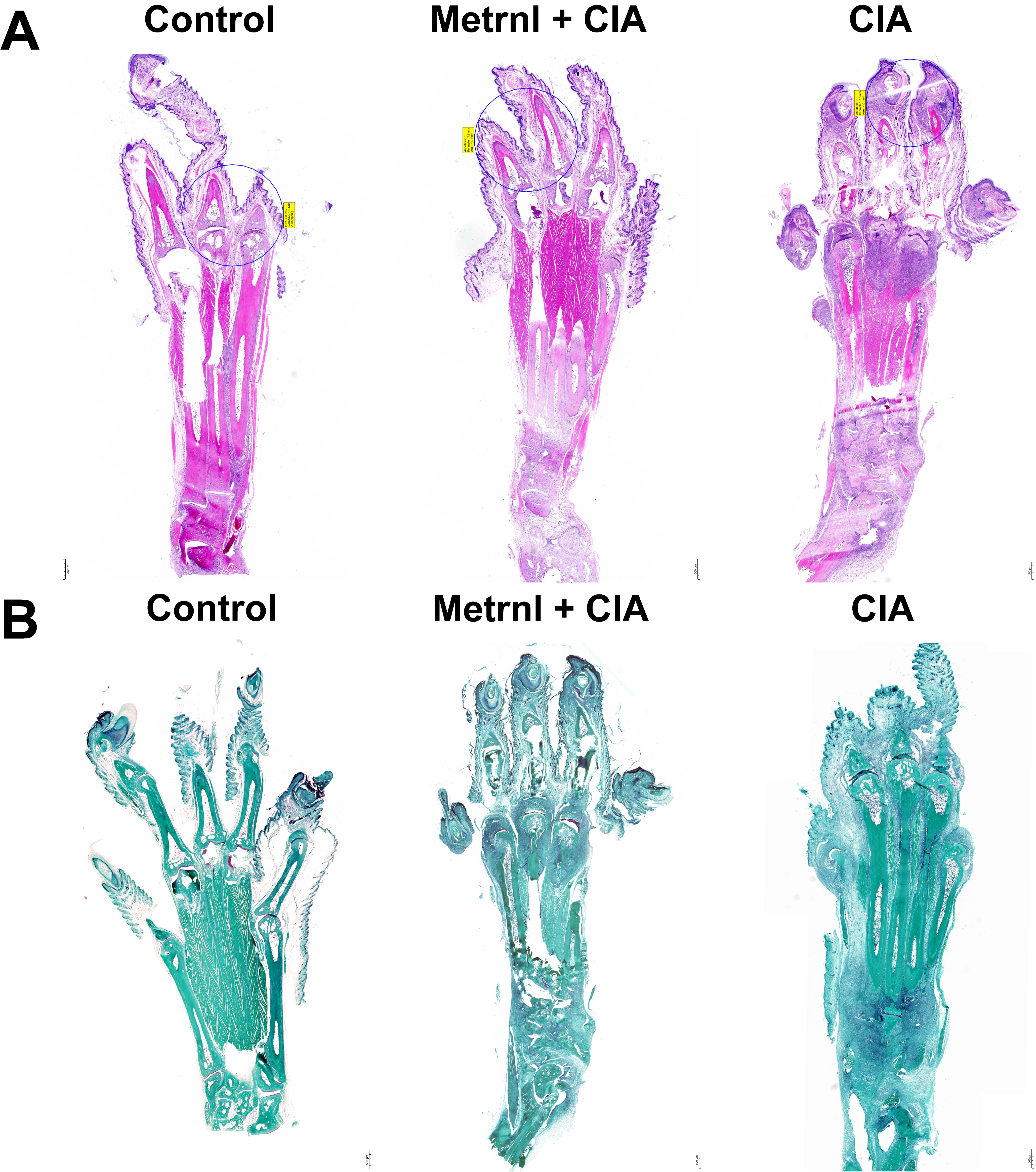


Figure S3. HE and Safranin O-Fast green staining of limbs.
